# Integrating genetically predicted transcriptomic signatures with longitudinal real-world data enables scalable drug repurposing for Alzheimer’s disease

**DOI:** 10.21203/rs.3.rs-9518587/v1

**Published:** 2026-05-15

**Authors:** Monika E. Grabowska, Rui Chen, Ying Zhou, Avi U. Vaidya, Xue Zhong, Chris Guardo, Alyson L. Dickson, Mojgan Babanejad, Chao Yan, Yi Xin, Sergio Mundo, Josh F. Peterson, Lang Li, Peter Embí, QiPing Feng, James Eaton, Zhexing Wen, Bingshan Li, Wei-Qi Wei

**Affiliations:** Vanderbilt University Medical Center; Vanderbilt University; Emory University School of Medicine; Vanderbilt University Medical Center; Vanderbilt University Medical Center; Vanderbilt University Medical Center; Vanderbilt University Medical Center; Vanderbilt University Medical Center; Vanderbilt University Medical Center; Vanderbilt University Medical Center; Vanderbilt University Medical Center; Vanderbilt University Medical Center; Ohio State University; Vanderbilt University Medical Center; Vanderbilt University Medical Center; Vanderbilt University Medical Center; Emory University School of Medicine; Vanderbilt University; Vanderbilt University Medical Center

## Abstract

Drug repurposing offers a potential strategy to expand treatment options for conditions with limited therapies, but advancing repurposing candidates toward clinical implementation remains a challenge. Large-scale data, together with advanced genetic and epidemiological methods, may help address this gap. Here, we present an integrative digital medicine approach that combines genetically predicted transcriptomic signatures and perturbation screening for candidate identification with multi-cohort real-world validation for systematic evaluation of prioritized candidates. We applied this approach to Alzheimer’s disease (AD), a disease with substantial unmet clinical need and persistent difficulty in developing effective therapies. We constructed AD signatures from genetically predicted expression changes across bulk tissues and microglia, then queried Connectivity Map profiles to identify compounds predicted to oppose these signatures. Aspirin emerged as a reproducible candidate across multiple signatures and underwent further evaluation. We then examined its association with incident AD in longitudinal electronic health record data from Vanderbilt University Medical Center and the NIH *All of Us* Research Program, as well as national insurance claims data. Across independent cohorts, aspirin initiation before age 65 was consistently associated with lower risk of incident AD, with signals suggesting that cumulative exposure and *APOE* ε4 status may influence effect size. Transcriptomic analysis of human cortical organoids provided additional experimental support, showing that aspirin more strongly opposed AD-related neuronal pathway alterations in wild-type organoids than in an organoid model of AD. This integrative approach offers a scalable strategy for genetically informed drug repurposing that bridges candidate discovery and clinical evaluation.

## Introduction

Many serious diseases still lack effective therapies despite substantial investment in drug development. Drug repurposing offers a practical complement to de novo drug development by identifying new uses for existing drugs. However, many repurposing studies stop at candidate nomination, creating a bottleneck in determining which of the many proposed candidates warrant further study^1^. Scalable approaches that integrate large-scale data, human genetics, and epidemiological methods can help move drug repurposing beyond candidate identification toward systematic evaluation, with the potential to accelerate clinical translation and improve patient outcomes.

Transcriptomic signature reversal has become a widely used strategy for drug repurposing, supported by the growing availability of large-scale genetic and transcriptomic datasets. This approach queries perturbational gene expression resources, such as the Connectivity Map (CMap), to identify compounds predicted to reverse disease-associated transcriptional profiles^2^. While early implementations often derived disease signatures from RNA sequencing or microarray data, transcriptome-wide association study (TWAS) methods offer a distinct genetics-informed approach by integrating expression quantitative trait loci (eQTLs) with genome-wide association study (GWAS) data to identify genes whose genetically predicted expression is associated with disease risk^3^. Because TWAS leverages germline genetic variation fixed at conception, it is less susceptible to reverse causation and may better prioritize causal genes and therapeutic targets. Given evidence that genetically supported drug targets are approximately 2.6 times more likely to succeed in clinical development^4^, TWAS-based signature reversal has emerged as a promising strategy for drug discovery and repurposing in complex diseases and has been applied to endometrial cancer, hypertension, and hyperlipidemia^5,6^.

After candidate identification, additional validation is needed to confirm preliminary signals and distinguish the most promising candidates. Real-world clinical data, including electronic health records (EHRs) and claims data, contain longitudinal information on medication exposures and clinical outcomes at scale and are increasingly used to generate and assess repurposing hypotheses in human populations^7-10^. This is especially relevant for conditions with a long preclinical phase, such as Alzheimer’s disease (AD), as candidate effects may depend on exposure timing and duration. Alongside clinical data, experimental studies in cellular models, such as human induced pluripotent stem cell (iPSC)-derived organoids, can provide complementary biological support for candidate assessment. Given the time and cost of downstream prospective clinical studies, integrating these sources of evidence is critical for directing resources toward the most compelling candidates.

In this study, we developed a genetics-informed repurposing workflow that integrates TWAS-derived disease signatures, perturbational screening, and longitudinal real-world clinical data. We then applied this workflow to AD. First, we identified AD-associated genes using GWAS summary statistics and eQTL data from bulk tissues and primary microglia, and used these genes to construct cross-tissue and microglia-specific disease signatures. We then queried CMap profiles to identify compounds opposing these signatures. Aspirin emerged as a recurrent candidate across multiple signatures and was subsequently evaluated in longitudinal EHR data from Vanderbilt University Medical Center (VUMC) and the National Institutes of Health *All of Us* Research Program, as well as in national claims data from the MarketScan Research Databases. We further examined aspirin-induced transcriptional responses in human iPSC-derived cortical organoids with wild-type and AD-associated APP mutant genotypes. Our findings illustrate how genetically informed candidate prioritization can be coupled with validation, including longitudinal clinical evaluation and experimental investigation, to advance drug repurposing and accelerate clinical implementation.

## Results

### Genetically informed Alzheimer’s disease transcriptomic signatures

**Figure 1:**
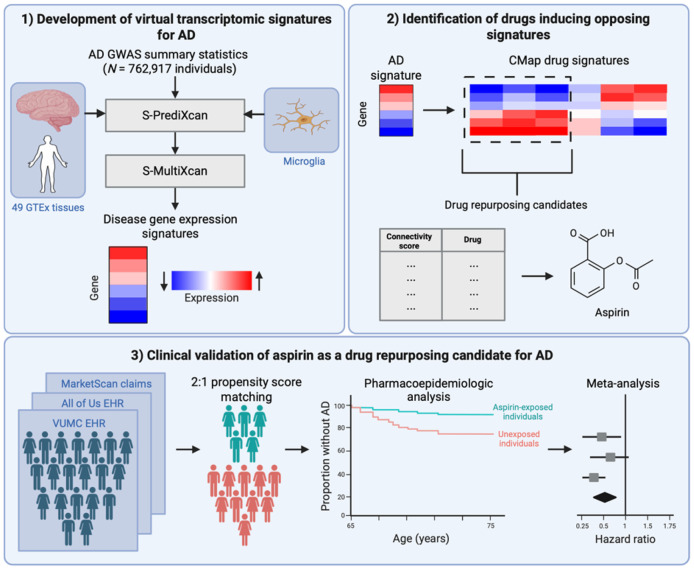
Integrative drug repurposing framework for Alzheimer’s disease. 1) Signature development: genetically informed AD signatures across 49 GTEx tissues and primary human microglia were derived from GWAS summary statistics using the S-PrediXcan and S-MultiXcan transcriptome-wide association study methods. 2) Candidate identification and prioritization: these AD signatures were queried against Connectivity Map (CMap) perturbational profiles to identify compounds predicted to reverse AD-associated expression changes. 3) Clinical validation: the leading candidate, aspirin, was evaluated using pharmacoepidemiologic analyses in longitudinal real-world clinical data, including electronic health record (EHR) data from Vanderbilt University Medical Center (VUMC), EHR data from the *All of Us* Research Program, and healthcare claims data from the MarketScan Research Databases.

An overview of the study design is shown in Fig. 1. Because the compounds returned by CMap depend directly on the disease signature used for querying, we constructed multiple TWAS-derived AD signatures spanning both bulk tissues and microglia rather than relying on a single transcriptomic profile.

We performed TWAS using S-PrediXcan^11^ and S-MultiXcan^12^ to identify candidate AD risk genes. Using pre-trained transcriptome prediction models for 49 tissues from the Genotype-Tissue Expression (GTEx) project^13^ and summary statistics from a large AD GWAS^14^, we conducted three S-MultiXcan analyses to balance tissue specificity and statistical power: (1) a brain-specific analysis combining the 13 GTEx brain tissues, (2) an AD-relevant tissue analysis with the 13 brain tissues plus four peripheral tissues previously related to AD (whole blood, spleen, and sun-exposed and unexposed skin)^15,16^, and (3) an all-tissue analysis combining all 49 GTEx tissues. AD risk genes were defined within each S-MultiXcan analysis as Bonferroni-significant TWAS associations (*P*<0.05/number of tested gene associations) and are reported in Supplementary Data File 1 (sheets 1-3).

**Figure 2:**
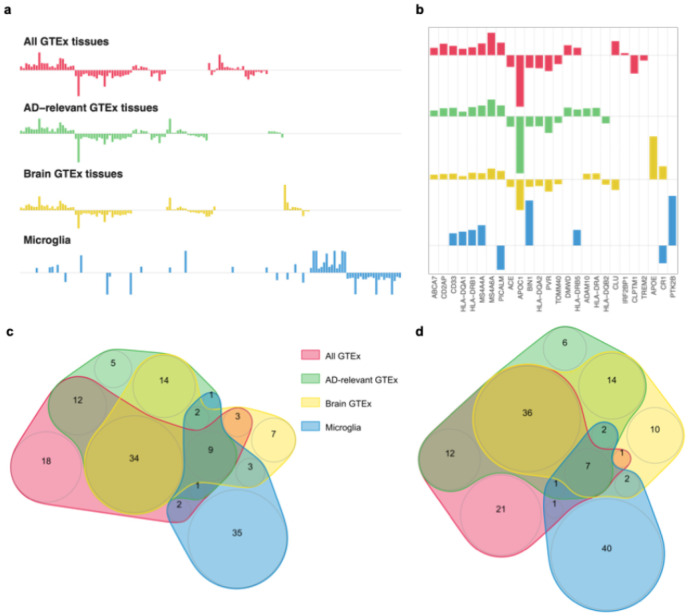
Genetically informed Alzheimer’s disease signatures across tissues and microglia. **a)** Genes included in the TWAS-derived AD signatures from all GTEx tissues, AD-relevant GTEx tissues, brain GTEx tissues, and microglia. Each vertical bar represents an individual gene, with the direction indicating a positive (upward) or negative (downward) association with AD risk. **b)** Associations between genetically predicted expression and AD risk for selected AD-relevant genes. **c)** Overlap of genes across the GTEx- and microglia-derived AD signatures irrespective of direction of association. **d)** Overlap of genes shared across signatures with concordant direction of association with AD risk.

We then constructed three GTEx-derived AD transcriptomic signatures (brain, AD-relevant, and all tissues) using the gene inclusion criteria described in Methods. The final brain-tissue AD transcriptomic signature contained 72 genes (37 positively associated with AD risk, 35 inversely associated), while the AD-relevant tissue signature contained 78 genes (41 positively, 37 inversely associated) and the all-tissue signature contained 79 genes (40 positively, 39 inversely associated) (Fig. 2a). A shared set of 43 genes was observed across all three GTEx signatures (21 positively associated with AD risk, 22 inversely associated).

Because bulk tissues can obscure cell type-specific signals and microglia play a central role in AD, we also constructed a microglial AD signature by performing TWAS with custom transcriptome prediction models trained on microglial eQTL summary statistics from the Microglia Genomic Atlas (MiGA)^17^. This signature comprised 53 genes (25 positively associated with AD risk, 28 inversely associated) (Fig. 2a). MiGA microglial TWAS results are provided in Supplementary Data File 1, sheet 4.

Across the four signatures, we identified genes previously implicated in AD, including *APOE*, *TREM2*, and *BIN1* (Fig. 2b; Supplementary Table 1). An annotated comparison of the four AD transcriptomic signatures is shown in Supplementary Fig. 1. Nine genes were shared across all four signatures (Fig. 2c), of which seven had directionally concordant associations with AD risk (Fig. 2d).

### Signature reversal prioritizes aspirin for downstream evaluation

We queried the Connectivity Map (CMap)^18^ using each TWAS-derived AD signature to identify compounds predicted to reverse disease-associated gene expression changes. Genes were assigned to up and down sets by TWAS *Z*-score sign. Compounds with negative connectivity scores (τ<0) were considered repurposing candidates^6,19^. Out of 2,428 small-molecule compounds in CMap, 590 (24.3%), 709 (29.2%), and 688 (28.3%) had negative connectivity to the GTEx brain-tissue, AD-relevant tissue, and all-tissue signatures, respectively (Supplementary Data File 1, sheets 5-7). Of these, 218 showed negative connectivity to all three GTEx signatures, including mycophenolate, fluticasone, sirolimus, sertraline, clozapine, and losartan, although connectivity magnitude varied widely (e.g., mycophenolate τ: brain-tissue −72.2; AD-relevant tissue −7.6; all-tissue −0.6). Additionally, 794 (32.7%) compounds had negative connectivity to the microglia-specific signature (Supplementary Data File 1, sheet 8). The top ten repurposing candidates for each signature are shown in Table 1.

The number and ranking of negatively connected compounds varied substantially across signatures, underscoring the importance of cross-signature support in candidate selection. Aspirin emerged as a recurrent candidate across multiple signatures, ranking among the top ten compounds for the all-tissue GTEx AD signature (τ=−69.96), with negative connectivity also observed for the AD-relevant GTEx tissue signature (−17.89) and the MiGA microglial signature (−30.88), although no connectivity was detected for the GTEx brain-tissue signature. For downstream evaluation, we focused on compounds showing negative connectivity across more than one AD signature and practical feasibility for longitudinal clinical evaluation. Aspirin met these criteria, with support across three signatures, widespread clinical use, and a well-characterized safety profile.

### Real-world clinical validation across three independent databases

We evaluated the prioritized aspirin signal in three real-world datasets: (1) VUMC’s de-identified EHR database, (2) the NIH *All of Us* Research Program database, and (3) the MarketScan Research Databases. In EHR data from VUMC and *All of Us*, we used a retrospective cohort study design to compare AD incidence after age 65 between aspirin-exposed patients (≥1 year of aspirin use before age 65) and propensity score-matched unexposed patients. In MarketScan claims, shorter follow-up limited ascertainment of incident AD among individuals with medication exposures documented before age 65; therefore, we used a case-control design comparing prior aspirin exposure in AD cases versus propensity score-matched controls. Descriptive characteristics for matched EHR cohorts are shown in Table 2. The characteristics of the matched MarketScan claims-based cohort are provided in Supplementary Table 2. Information on AD outcomes in all three datasets is provided separately in Supplementary Table 3.

**Figure 3:**
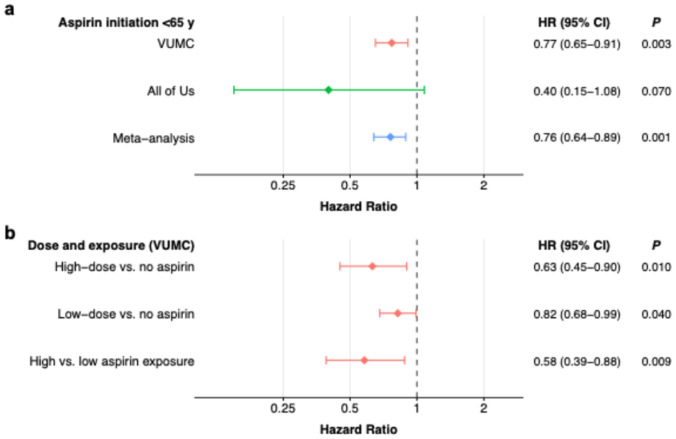
Longitudinal real-world clinical evaluation of aspirin. **a)** Associations between aspirin initiation before age 65 and incident AD after age 65 in electronic health record (EHR) data from Vanderbilt University Medical Center (VUMC) and the *All of Us* Research Program, including pooled estimates from metaanalysis. Forest plots show hazard ratios (HRs) with 95% confidence intervals (CIs); the dashed vertical line indicates the null (HR=1). b) Dose- and exposure-stratified analyses within the VUMC cohort. Comparisons evaluate high-dose (≥325mg/day) and low-dose (≤81mg/day) aspirin use versus no aspirin use, and high versus low documented aspirin exposure rate. Documented aspirin exposure rate was used as a proxy for cumulative aspirin exposure, with high exposure defined as above the cohort median of 5 records/year.

In VUMC, aspirin use before age 65 was associated with a significantly reduced risk of incident AD after age 65 (hazard ratio [HR]=0.77, 95% confidence interval [CI]: 0.65-0.91, *P*=0.003; Fig. 3a). In *All of Us*, the association was directionally similar but limited by low statistical power (HR=0.40, 95% CI: 0.15-1.08, P=0.07; *N*=24 AD events among 1,995 participants). Meta-analysis of the two EHR cohorts showed that aspirin initiation before age 65 was associated with 24% lower risk of incident AD (HR=0.76, 95% CI: 0.64-0.89, *P*=0.001; Fig. 3a). In MarketScan, patients diagnosed with AD were less likely to have prior aspirin exposure compared to matched controls (OR=0.32, 95% CI: 0.28-0.38, *P*<0.001).

We performed secondary analyses in VUMC to examine whether the association varied by aspirin dose or cumulative exposure. Due to the limited number of AD events in the *All of Us* cohort and the small number of aspirin prescriptions in MarketScan, we were unable to evaluate these measures in those datasets. In dose-stratified analyses (high-dose ≥325mg/day; low-dose ≤81mg/day^20^), aspirin use remained associated with lower AD risk for both high-dose (HR=0.63, 95% CI: 0.45-0.90, *P*=0.01) and low-dose regimens (HR=0.82, 95% CI: 0.68-0.99, *P*=0.04), with no significant difference between dose groups (*P*=0.19). Because higher aspirin doses may reflect more severe underlying indications, we also examined cumulative exposure. Given inconsistent documentation of dosing frequency and treatment duration in the EHR, we used documented aspirin exposure rate, defined as the number of unique aspirin records divided by the years between first and last recorded exposure, as a proxy. Exposure rates above the cohort median (>5/year) were associated with lower AD risk (HR=0.58, 95% CI: 0.39-0.88, *P*=0.009; Fig. 3b).

In addition, we conducted *APOE*-stratified analyses in VUMC and *All of Us* (MarketScan does not contain genetic data). Among *APOE* ε4 carriers, aspirin use before age 65 showed a suggestive inverse association with incident AD in both cohorts, although neither analysis reached statistical significance individually (VUMC HR=0.60, 95% CI: 0.33-1.10, *P*=0.0986; *All of Us* HR=0.48, 95% CI: 0.10-2.25, *P*=0.35). Combined meta-analysis showed a 41% decreased risk of incident AD after age 65 in *APOE* ε4 carriers (HR=0.59, 95% CI: 0.33-1.02, *P*=0.06), suggesting a potentially stronger protective association in this subgroup but limited by statistical power. Aspirin use was not significantly associated with decreased AD risk among non-carriers (VUMC HR=0.63, 95% CI: 0.26-1.55, *P*=0.317; *All of Us* HR=0.52, 95% CI: 0.15-1.79, *P*=0.3; meta-analysis HR=0.59, 95% CI: 0.29-1.22, *P*=0.16), although the power of these analyses was limited by the small number of AD cases.

### Transcriptomic evaluation in human iPSC-derived cortical organoids

To assess the biological plausibility of the aspirin signal, we treated 90-day-old cortical organoids derived from isogenic control wild-type (WT; WTC11) and heterozygous APP mutant (KM670/671NL) iPSCs with aspirin (0.5 mM) or vehicle (PBS) for one week, followed by RNA sequencing (RNA-seq). High uniquely mapped paired-end rates (~90% across samples) indicated successful library preparation and sequencing (Supplementary Data File 1, sheet 9). Replicates showed high concordance and clustered cleanly by their assigned labels (Supplementary Fig. 2), supporting the expected data quality.

Over-representation analysis of differentially expressed genes (∣log2FC∣ ≥log2(1.1), FDR<0.05) highlighted synapse- and axon-related pathways, including glutamatergic synapse and axon guidance, in both the baseline APP mutation signature and the WT aspirin response (Supplementary Fig. 3a). Gene set enrichment analysis (GSEA) showed broad downregulation of neuronal and synaptic terms in the baseline APP signature, including synaptic signaling (NES=−2.29, FDR=4.11×10^−31^) and axon development (NES=−2.11, FDR = 2.76×10^−15^) (Supplementary Fig. 3b). In WT organoids, aspirin produced the opposite pattern, with strong upregulation of synaptic signaling (NES=2.52, FDR=8.16×10^−48^) and axon development (NES=2.56, FDR=3.75×10^−39^) (Supplementary Fig. 3c). In contrast, aspirin-treated APP mutant organoids showed attenuated enrichment of neuronal terms relative to WT, with top GSEA hits predominantly reflecting cell cycle and chromosome-associated processes (Supplementary Fig. 3d). Full GSEA results for all contrasts are provided in Supplementary Data File 1, sheets 10-12.

**Figure 4:**
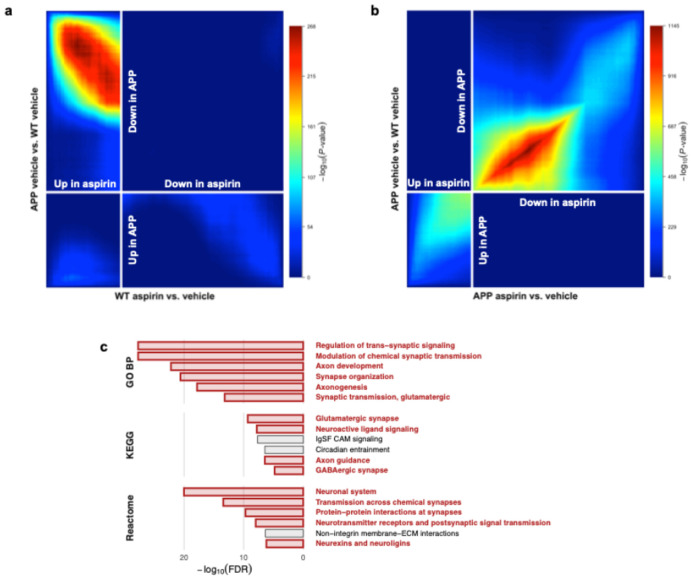
Rank-rank hypergeometric overlap between aspirin-induced transcriptional changes and the baseline APP mutation signature. **a)** Rank-rank hypergeometric overlap (RRHO) heatmap comparing the aspirin response in wild-type organoids (WT aspirin vs. vehicle) with the baseline APP mutation signature (APP vehicle vs. WT vehicle). The strongest discordant overlap is observed among genes downregulated in APP and upregulated by aspirin in WT (upper left quadrant). **b)** RRHO heatmap comparing the aspirin response in APP mutant organoids (APP aspirin vs. vehicle) with the baseline APP mutation signature, showing predominantly concordant overlap (down-down in the upper right quadrant, up-up in the lower left quadrant). For RRHO, genes were ranked from most upregulated to most downregulated using - log_10_(*P*) × sign(log2 fold change). Heatmap intensity reflects overlap significance across rank thresholds, shown as −log_10_(*P*) from the hypergeometric test, with warmer colors indicating stronger overlap. **c)** Functional enrichment of the discordant genes from panel a (up in WT aspirin, down in APP). The top five enriched gene sets per database (GO BP, KEGG, Reactome) are shown; bars denote - log_10_(FDR). Gene sets related to neuronal signaling and organization are bolded and outlined in red.

In WT organoids, the transcriptional effects of aspirin were modestly but significantly negatively correlated with the baseline APP mutation signature (Spearman’s ρ=−0.15, *P*<2.2×10^−16^, *N*=30,848 genes), consistent with partial reversal of disease-associated changes. Rank-rank hypergeometric overlap (RRHO) analysis^21^ identified a discordant hotspot comprising genes upregulated by aspirin in WT organoids and downregulated in the baseline APP signature (Fig. 4a). In APP mutant organoids, the aspirin response was positively correlated with the APP signature (ρ=0.37, *P*<2.2×10^−16^, *N*=30,848 genes), and RRHO showed a dominant concordant overlap signal (Fig. 4b), providing little evidence of global transcriptomic reversal in the APP background. Genes in the discordant hotspot in Fig. 4a were enriched for synaptic signaling and neurotransmission across GO Biological Process, KEGG, and Reactome databases, with top terms including regulation of trans-synaptic signaling, glutamatergic and GABAergic synapse, and transmission across chemical synapses (Fig. 4c; Supplementary Data File 1, sheet 13).

**Figure 5:**
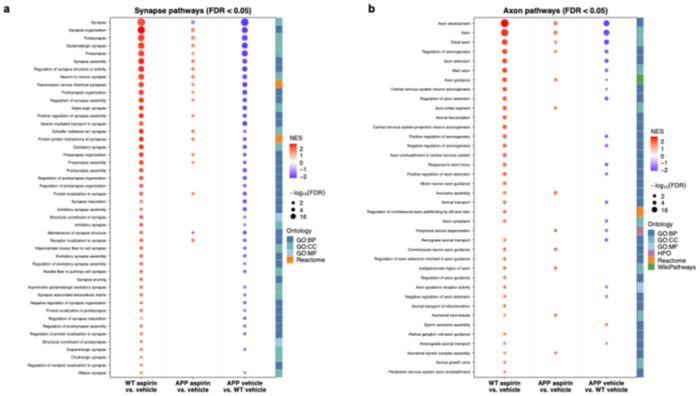
Synapse- and axon-related pathway enrichment across aspirin-treated wild-type and APP mutant organoids. **a)** Gene set enrichment results for synapse-related MSigDB gene sets across three contrasts: wild-type (WT) aspirin versus vehicle, APP aspirin versus vehicle, and APP vehicle versus WT vehicle. **b)** Gene set enrichment results for axon-related MSigDB gene sets across the same contrasts. Only pathways with false discovery rate (FDR) <0.05 are shown. Bubbles are colored by normalized enrichment score (NES), with red indicating positive enrichment and blue indicating negative enrichment. Bubble size reflects statistical significance, shown as −log_10_FDR. Across both pathway families, aspirin induced stronger reversal of the baseline disease-associated pattern in WT than in APP mutant organoids.

Building on these findings, we performed targeted GSEA of synapse-, axon-, and neurotransmitter-related gene sets curated from MSigDB using keyword filters. The baseline disease signature (APP vehicle vs. WT vehicle) showed broad downregulation across these pathway families, consistent with neuronal dysfunction in AD. In WT organoids, aspirin robustly upregulated these pathways, counteracting the baseline AD signature (synapse and axon pathways in Fig. 5 and Supplementary Figs. 4-5; neurotransmitter pathways in Supplementary Fig. 6). By contrast, in APP mutant organoids, aspirin’s effects were attenuated and did not consistently oppose the baseline disease signature.

## Discussion

We developed an integrative drug repurposing framework that combines genetically informed transcriptomic disease signatures, perturbational signature matching, longitudinal real-world clinical data, and experimental investigation in human cellular models to prioritize candidates for further study and accelerate clinical translation. Applying this framework to AD, we identified aspirin as a candidate supported across multiple disease signatures, three independent clinical datasets, and transcriptomic analyses in human cortical organoids. This study illustrates how genetically informed candidate prioritization can be paired with large-scale longitudinal clinical data to evaluate repurposing hypotheses in a scalable and clinically relevant manner.

An important feature of this study is the use of multiple disease signatures spanning both bulk tissues and microglia rather than a single transcriptomic representation of AD. Our TWAS analyses highlighted genes at established AD risk loci, including *APOE*, *TREM2*, and *BIN1*, and corroborated previously reported TWAS associations (Supplementary Table 1), while also revealing substantial variability across tissues and cell types. Only seven AD risk genes appeared in all four signatures with concordant effect directions. Notably, *BIN1*, a leading late-onset AD risk locus, showed a positive association with AD risk in microglia but inverse associations in all GTEx signatures. This variability was also reflected in the top-ranked CMap compounds identified across the four TWAS-derived AD signatures, supporting the use of cross-signature consistency as a prioritization criterion. We therefore focused on compounds with negative connectivity across more than one AD signature and practical feasibility for longitudinal clinical evaluation. Aspirin met these criteria, with support across the all-tissue GTEx, AD-relevant GTEx, and microglial signatures, making it a useful test case for this framework.

Across three real-world datasets, aspirin exposure was consistently associated with lower AD risk. In meta-analysis of the two EHR cohorts, aspirin initiation before age 65 was associated with a 24% reduced risk of incident AD. The claims-based study, while constrained by shorter observation windows that precluded time-to-event analysis, showed directionally consistent results in a case-control analysis. We did not detect a significant difference between high- and low-dose regimens, but individuals with higher documented aspirin exposure rates, used here as a proxy for cumulative exposure, had lower AD risk than matched individuals with lower exposure rates. This observation is consistent with UK Biobank analyses in which the inverse association between low-dose aspirin and AD was most evident with long-term use (>10 years)^22^. Analysis of EHR-linked genetic data suggested a stronger inverse association among individuals carrying at least one *APOE* ε4 allele, although statistical power was limited by the relatively small number of *APOE* ε4 carriers and low prevalence of AD diagnoses among non-carriers. Overall, these findings support the consistency of the aspirin signal across distinct clinical data sources and illustrate how longitudinal clinical data can refine repurposing signals beyond simple exposed-versus-unexposed comparisons.

Transcriptomic analysis of human iPSC-derived cortical organoids provided complementary experimental support for the aspirin signal. In wild-type organoids, aspirin-induced transcriptional changes were directionally opposite to the APP mutation signature and were enriched for synaptic, axonal, and neurotransmission-related pathways suppressed in the baseline disease state. In contrast, aspirin responses in APP mutant organoids were attenuated and did not show the same degree of opposition to the baseline disease signature. These findings provide biological plausibility for the prioritized signal and suggest that aspirin’s effects may depend on underlying disease context.

Randomized trials have not demonstrated a clear population-level benefit of aspirin in AD, although most initiated treatment relatively late in life^23-26^, after AD-related pathology may already have been established^27,28^. Our findings do not contradict those trials; rather, they raise the possibility that any benefit of aspirin, if present, may depend on exposure timing and persistence during the long preclinical interval preceding diagnosis, when relevant neuronal pathways may still be modifiable. This interpretation is consistent with the genetically anchored, life-course nature of TWAS-derived signatures, and with our real-world analyses, which specifically emphasized aspirin initiation before age 65. It is also broadly concordant with the organoid results, where aspirin more strongly opposed disease-associated neuronal pathway alterations in wild-type than APP mutant backgrounds, suggesting reduced efficacy in a disease context.

This study has several strengths. It integrates human genetics, perturbational screening, and multi-cohort real-world clinical data within a single repurposing workflow and evaluates the leading candidate across independent EHR and claims datasets, as well as through transcriptomic analysis in human iPSC-derived organoids. Using TWAS, we mapped AD genetic risk to genetically predicted gene expression across multiple tissues and in microglia, generating disease-relevant signatures for candidate identification. Longitudinal EHR data enabled evaluation of aspirin initiation over intervals that are difficult to study in conventional trials and supported additional analyses by exposure pattern and *APOE* ε4 status. Importantly, this framework is transferable to other complex diseases with available GWAS summary statistics and longitudinal EHR or claims data, where genetics-informed prioritization can be integrated with real-world data to support more systematic assessment of repurposing candidates.

This study also has several limitations. Repurposing hypotheses were restricted to compounds profiled in CMap, which is not exhaustive and is derived largely from non-brain cell lines that may not fully reflect AD-relevant biology. TWAS was constrained by the ancestral composition of available AD GWAS and eQTL reference datasets, and we lacked power for ancestry-stratified EHR analyses despite known differences in AD incidence among populations, which may limit generalizability. AD case definitions relied on structured diagnosis codes and may incompletely capture clinical heterogeneity and potentially introduce outcome misclassification. Aspirin exposure was also likely under-ascertained because over-the-counter use is not represented in pharmacy claims and is inconsistently recorded in EHR medication lists. These outcome and exposure measurement limitations would both be expected to attenuate associations toward the null. Although we addressed measured confounding through propensity score matching, residual confounding remains possible. Finally, organoids lack systemic vascular and immune context, precluding assessment of aspirin’s antithrombotic and peripheral anti-inflammatory effects that are likely relevant *in vivo*.

The workflow described here provides a scalable strategy for moving from computationally prioritized repurposing signals to clinical evaluation in longitudinal real-world data, particularly relevant for diseases in which conventional drug development is slow, costly, or poorly aligned with disease stage. Given the flexibility of this workflow, future updates could incorporate additional identification or validation steps, such as AI-facilitated screening^10^. In AD, our findings support further study of aspirin with earlier initiation rather than broad late-life use. Future observational studies incorporating deeper phenotyping, including neuroimaging, cerebrospinal fluid biomarkers, polygenic risk scores, and cardiovascular and metabolic risk measures, may help define the subgroups most likely to benefit. Overall, this approach demonstrates a scalable next step for advancing drug repurposing toward eventual clinical implementation.

## Methods

This study was conducted with approval from the VUMC Institutional Review Board and the NIH *All of Us* Research Program. All EHR data from VUMC and *All of Us* are de-identified; use of these data is considered non-human subjects research.

### Construction of microglia transcriptome prediction models

We downloaded full nominal eQTL summary statistics for 255 primary human microglia samples across four brain regions (medial frontal gyrus, superior temporal gyrus, subventricular zone, thalamus) from 100 human subjects from the Microglia Genomic Atlas (MiGA)^17^. Details on genotyping, RNA-seq generation and processing, and eQTL mapping can be found in the MiGA flagship paper^29^. Using the multivariate adaptive shrinkage approach to eQTL analysis introduced by Urbut *et al*.^30^, we trained region-specific Multivariate Adaptive Shrinkage in R (MASHR) transcriptome prediction models. For each brain region, we selected the top five eQTLs with the lowest local false sign rate (a measure analogous to false discovery rate)^30^ per gene and included these eQTLs in the final models. In cases where multiple eQTLs were tied for lowest local false sign rate, we retained all eQTLs. An initial top-eQTL-per-gene specification was evaluated but not used in downstream analyses due to limited overlap with the AD GWAS variant set.

### Imputation of transcriptomic signatures for Alzheimer’s disease

We applied two statistical methods, S-PrediXcan^11^ and S-MultiXcan^12^, to publicly available Alzheimer’s GWAS summary statistics to compute transcriptomic signatures for AD. S-PrediXcan and S-MultiXcan predict gene expression for a trait or disease of interest using prediction models trained on reference transcriptome datasets. S-PrediXcan computes single-tissue gene-level association results from GWAS summary statistics. S-MultiXcan then aggregates the single-tissue S-PrediXcan results across multiple tissues, thereby increasing the statistical power to detect associations. We used GWAS summary statistics for a total of 762,917 individuals (86,531 AD cases and 676,386 controls, excluding participants from 23andMe, which were not available due to data access restrictions)^14^ to generate all AD transcriptomic signatures used in this study. To improve GWAS-QTL integration, we harmonized the AD GWAS summary statistics to GTEx v8 variants and imputed summary statistics for missing variants^31^.

We first used S-PrediXcan to impute single-tissue gene expression levels in 49 available GTEx tissues, using pre-developed MASHR expression prediction models^11,32,33^. We then ran S-MultiXcan on the S-PrediXcan results to predict gene expression across multiple tissues. Although AD predominantly affects the brain, studies have suggested that peripheral tissues such as the skin and vascular tissues can also capture genetic effects on gene expression in AD and may even represent potential pathogenic tissues in AD^15^. Thus, we conducted three separate S-MultiXcan analyses to predict AD-associated changes in gene expression combining brain and non-brain tissues, including: (1) a brain-specific analysis combining predictions from the 13 GTEx brain tissues, (2) an AD-relevant tissue analysis combining the 13 brain tissues with four other tissues previously related to AD (whole blood, spleen, and two skin tissues)^15,16^, and (3) a non-specific analysis combining predictions from all 49 GTEx tissues. We then constructed a separate AD virtual transcriptomic signature for each of the brain-restricted, AD-relevant, and all-tissue S-MultiXcan analyses using the AD risk genes with ≥2/3 tissue concordance (i.e., showing the same directionality of gene expression changes in at least two-thirds of the tissues with non-NA results). We required this tissue-wide consensus because several genes with statistically significant S-PrediXcan results exhibited opposing effects across tissues, producing near-zero effect estimates when averaged in S-MultiXcan analysis (e.g., *APOE* in the all-tissue analysis, Supplementary Fig. 7). The tissue-specific S-PrediXcan results for the AD risk genes, their consensus direction of effect, and mean *Z* score computed across the tissues with the consensus effect direction are available in Supplementary Data File 1, sheets 1-3. We defined AD risk genes using Bonferroni correction (*P*<0.05/number of S-MultiXcan gene associations). We used the mean *Z* score among the concordant tissues to classify the risk genes as positively associated with AD risk (mean *Z* score>0) or inversely associated with AD risk (mean *Z* score<0).

We also used S-PrediXcan and S-MultiXcan to calculate a microglia-specific AD gene expression signature using the microglia MASHR transcriptome prediction models we trained with MiGA data. We then applied S-PrediXcan to predict microglial gene expression in the medial frontal gyrus, superior temporal gyrus, subventricular zone, and thalamus. We initially used S-MultiXcan to construct a single AD gene expression signature integrating the S-PrediXcan results for microglia in the four different brain regions; however, the high correlations between the S-PrediXcan associations across the brain regions prevented computation of S-MultiXcan association statistics for >80% (7,852/9,687) of the genes tested. We thus used a generalized Berk-Jones (GBJ) test to combine the S-PrediXcan *Z* scores from the four brain regions^34^. Again, AD risk genes were defined using a Bonferroni-corrected significance threshold (GBJ *P*<0.05/18,222 genes tested). As GBJ does not provide directional effects (GBJ statistic is always positive), we constructed the microglial signature including only the AD risk genes with concordant effect directions across all regions with available results. NA values did not influence the concordance assessment. To capture inverse associations with AD risk, we negated the GBJ statistic for genes with consistently negative S-PrediXcan *Z* scores across the brain regions.

### Identification of drug repurposing candidates

We queried the Connectivity Map (CMap) to identify drugs with perturbation profiles opposing TWAS-derived AD signatures. CMap contains over 1.5 million gene expression profiles (including 978 directly measured landmark genes) capturing the effects of over 8,000 small-molecule compounds and genetic reagents across multiple human cell lines^35^. Each AD signature was represented as two gene sets split by TWAS direction (*Z*>0 “up”; *Z*<0 “down”) and submitted to the CMap CLUE Query tool, which returns a connectivity score (τ) quantifying similarity between the queried signature and each perturbagen signature. Small-molecule compounds with τ<0 were considered potential repurposing candidates, consistent with prior CMap-based studies^6,19^. For downstream evaluation, we prioritized compounds with negative connectivity across multiple AD signatures, feasible exposure ascertainment in real-world data, and sufficient expected numbers of exposed individuals for longitudinal analysis.

### Clinical evaluation using real-world data

To evaluate the prioritized aspirin signal in longitudinal clinical data, we investigated the association between aspirin exposure and AD using EHR and claims data. In the two EHR databases, we performed a retrospective cohort study evaluating the risk of new AD diagnosis after age 65 in individuals previously exposed to aspirin compared to individuals without documented exposure. In the claims database, we performed a case-control study comparing the odds of prior aspirin use in AD cases and controls.

#### Data sources

We performed clinical validation studies using diagnosis and medication data from three independent sources: (1) VUMC’s Synthetic Derivative (SD) database, (2) the NIH *All of Us* Research Program database, and (3) the MarketScan Commercial Claims and Encounters (CCAE) and Medicare Supplemental (MDCR) databases. The VUMC SD contains decades of longitudinal clinical data, including diagnosis and procedure codes, laboratory test results, and medications, extracted from de-identified EHRs for over 4 million unique patients^36^. The SD is linked to VUMC’s biobank, BioVU, which contained over 300,000 unique DNA samples as of January 2023, allowing for the integration of EHR and genetic data. At the time of the study, the *All of Us* Research Program database contained data for over 633,540 participants, with genomic sequencing data for over 414,840 participants^37^. The MarketScan CCAE and MDCR databases collectively contain detailed insurance claims data, including diagnosis codes and pharmacy claims, for over 200 million unique patients^38^.

#### Cohort formation and outcome assessment: VUMC and All of Us

To capture endpoints relevant to AD and ensure adequate EHR follow-up time was available for all patients in the study population, we restricted our analysis to patients over 65 years of age with at least one visit at ≥75 years. We excluded individuals with AD diagnosed at ≤65 years, as well as individuals with diagnoses of non-AD dementia (occurring at any time).

Information on patient medications is available as structured data in the VUMC and *All of Us* EHR databases (within a DRUG_EXPOSURE table, in accordance with the Observational Medical Outcomes Partnership Common Data Model^39^). Drug exposures documented in this table may originate from various sources in the EHR, including clinical notes (e.g., medication lists appearing in history and physical [H&P] reports, progress notes, and discharge summaries), inpatient and outpatient medication orders (e.g., prescriptions), and historical medication records.

We identified aspirin-exposed patients by mapping patient medications to their ingredients using the RxNorm standardized terminology for clinical drugs and filtering for all medications containing aspirin (RxCUI = 1191). We used aspirin-exposed patients with at least one year of documented aspirin use prior to age 65 to form the aspirin-exposed group; patients without any documented aspirin use were considered unexposed controls. Patients with first aspirin use occurring after age 65 were excluded. We then identified two groups within the aspirin-exposed cohort for additional comparisons based on the two most common doses at VUMC: high-dose (≥325mg/day) and low-dose (≤81mg/day) aspirin users, excluding individuals exposed to intermediate aspirin doses and individuals switching between low-dose and high-dose aspirin use.

Aspirin is used in the treatment of multiple medical conditions, including in doses of 81 and 325 mg for primary and secondary prevention of atherosclerotic cardiovascular disease^40^, and historically, in much higher doses for pain relief in inflammatory diseases such as rheumatoid arthritis^41^, which may also impact AD risk. To minimize the effects of confounding by indication and other sources of confounding, we matched patients in the aspirin-exposed cohort to control patients with no recorded aspirin use in a 1:2 ratio, using a propensity score based on sex, race, EHR time after age 65 (i.e., length of time between age 65 and last EHR record), and presence of clinical indications for aspirin use (cardiovascular disease, cerebrovascular disease, and rheumatoid arthritis) at baseline (defined as the time zero of age 65 or, if no data available at age 65, the age of the first EHR visit after 65). We used the MatchIt R^42^ package to perform nearest-neighbor propensity score matching. The diagnoses used to define the comorbidities for matching are provided in Supplementary Data File 1, sheets 14-16.

We defined AD cases using a requirement of at least one AD diagnosis code (ICD-9-CM 331.0; ICD-10-CM G30.1, G30.8, G30.9). We excluded patients with a first recorded AD code before age 65 and those with codes for non-AD dementias (Supplementary Data File 1, sheet 17).

#### Statistical analysis: VUMC and All of Us

We used Cox proportional hazards regression models to investigate incident AD risk after age 65 in aspirin-exposed and unexposed individuals. Age 65 served as time zero; individuals were followed until first recorded AD diagnosis or otherwise right censored at last recorded EHR observation. We first compared AD risk between the aspirin-exposed cohort and the propensity score-matched unexposed cohort. We then performed subgroup analyses based on aspirin dose (high-dose versus low-dose versus no aspirin), documented aspirin exposure rate, and *APOE* ε4 genotype.

We used the metafor R package for meta-analysis of hazard ratios^43^. Heterogeneity was assessed using Cochran’s Q and I^2^. Based on these metrics, all meta-analyses were conducted under a fixed-effects model.

#### Documented aspirin exposure rate

The inconsistent recording of medication end dates, dosing frequency (e.g., daily versus as needed), and therapy duration within the EHR, along with multiple sources of medication documentation in patient charts (including medication lists in clinical notes as well as prescriptions), hindered precise quantification of total aspirin exposure using EHR data. To address this limitation, we developed a proxy measure: the documented aspirin exposure rate, defined as the total number of unique aspirin records divided by the time (in years) between the first and last recorded aspirin exposures. This measure was intended to capture the frequency and duration of aspirin use documented in the EHR, with a higher rate reflecting more consistent and sustained aspirin exposure. We calculated the documented aspirin exposure rate for all individuals in the aspirin-exposed cohort and then classified aspirin users into high- and low-exposure groups based on the median documented exposure rate of 5 aspirin records per year. To ensure balanced comparisons, we matched individuals with high documented aspirin exposure rates to those with low rates in a 1:1 ratio using propensity score matching. The variables used in matching were sex, race, baseline comorbidities (cardiovascular disease, cerebrovascular disease, rheumatoid arthritis), EHR time after age 65, total number of EHR visits, and aspirin duration. The final matched cohort comprised 3,690 individuals (*N* = 1,845 per group). A Cox proportional hazards regression model was used to investigate the risk of AD after age 65 in the high-exposure group relative to the low-exposure group.

#### APOE genotyping

*APOE* genotype was determined using the combination of alleles at SNPs rs429358 and rs7412. The *APOE* ε4 variant was defined as the presence of a C allele at both SNPs. Genetic data was available for 1,856 patients in the VUMC cohort (including 41 APOE ε4 homozygotes and 430 heterozygotes) and 1,450 patients in the *All of Us* cohort (with 23 APOE ε4 homozygotes and 271 heterozygotes). Information on *APOE* genotype was not available in the MarketScan dataset.

#### MarketScan validation

Given the shorter observation time in the MarketScan Research Databases, which prevented us from capturing AD-relevant timepoints in patients exposed to aspirin before age 65, we performed a casecontrol study to investigate the association between aspirin use and AD. We first identified AD cases using ICD-9-CM code 331.0 and ICD-10-CM codes G30.1, G30.8, and G30.9. We matched AD cases to comparable controls in a 1:2 ratio based on propensity score, using sex, comorbidities (cardiovascular disease, cerebrovascular disease, and rheumatoid arthritis, diagnosed at any age), and claims follow-up time (difference in years between first and last claims records) as covariates. We did not match on race as this is not reported in MarketScan. Aspirin prescriptions were identified using National Drug Codes. We then calculated the odds ratio for aspirin exposure among the AD cases compared to their matched controls.

### Human iPSC culture and cortical organoid generation

Isogenic control wild-type (WT) and heterozygous APP mutant (KM670/671NL) iPSCs were maintained in mTeSR1 medium and passaged every 6-7 days. Human cortical organoids were generated following a previously established differentiation protocol, with modifications^44,45^. Briefly, iPSC colonies were enzymatically detached using 1 mg/mL collagenase IV for 1 hour. The iPSC colonies were collected and cultured as embryoid bodies (EBs) in DMEM/F12 medium (Invitrogen) supplemented with 20% Knockout Serum Replacement (KSR, Invitrogen), 1× GlutaMAX (Invitrogen), 1×MEM Non-Essential Amino Acids (Invitrogen), 0.1 mM beta-mercaptoethanol (Invitrogen), 2 µM Dorsomorphin (PeproTech), and 2 µM A-83 (PeproTech). EBs were maintained in 10 cm low-attachment dishes for 5 days, allowing uniform spheroid formation. From day 6 to day 16, EBs were changed into a neural medium (NM) comprising Neurobasal medium (Invitrogen), 1× B-27 supplement (minus vitamin A, Invitrogen), 1× GlutaMAX (Invitrogen), and 100 U/mL penicillin-streptomycin (Invitrogen), further supplemented with 20 ng/mL bFGF (Pepro Tech) and 20 ng/mL EGF (Pepro Tech). The medium was replaced daily to support robust neuroectodermal patterning. Between day 17 and day 24, cultures were maintained in the same medium with changes every other day. On day 25, the neural medium was supplemented with 20 ng/mL BDNF, with medium renewal continued every other day. By day 43, the medium was shifted to differentiation medium with growth factor-free neural medium, consisting of Neurobasal, 1× B-27 supplement (minus vitamin A), 1× GlutaMAX, and 100 U/mL penicillin-streptomycin, refreshed every four days. From day 70 onward, organoids were cultured in NM supplemented with 1× B-27 containing vitamin A, with medium changes every three days, promoting long-term maturation. At day 90, mature human cortical organoids were treated with acetylsalicylic acid (aspirin; Sigma A5376; 0.5 mM) or vehicle (PBS) for one week.

### RNA extraction and RNA-seq

Human cortical organoids were homogenized in TRIzol Reagent (Invitrogen, 15596018), and total RNA was extracted using the Direct-zol^™^ RNA Miniprep Kit (Zymo, R2052) according to the manufacturer’s instructions. Total RNA was quantified using the Qubit 2.0 Fluorometer (ThermoFisher Scientific, Waltham, MA, USA) and assessed for integrity with the 4200 TapeStation (Agilent Technologies, Palo Alto, CA, USA). Strand-specific libraries were prepared using the NEBNext Ultra II Directional RNA Library Prep Kit for Illumina (NEB, Ipswich, MA, USA), following the manufacturer’s instructions. RNA was fragmented at 94 °C for 8 minutes, and first- and second-strand cDNA synthesis was performed, with dUTP incorporated during second-strand synthesis to maintain strand specificity. After 3’ end adenylation, adapter ligation, and limited-cycle PCR amplification, libraries were validated using the Agilent TapeStation and quantified by Qubit 2.0 (ThermoFisher Scientific) and qPCR (KAPA Biosystems, Wilmington, MA, USA). Libraries were multiplexed, clustered onto a flowcell, and sequenced on the Illumina NovaSeq 6000 system using a 2 × 150 bp paired-end configuration, according to the manufacturer’s protocol. Image analysis and base calling were performed using the NovaSeq Control Software (Illumina, San Diego, CA, USA), and raw BCL files were converted to FASTQ format and demultiplexed with bcl2fastq v2.20 (Illumina), allowing one mismatch for index recognition.

### RNA-seq analysis

RNA-seq data were processed following a previously described workflow. Raw reads were quality-checked to confirm that library preparation and sequencing met requirements for downstream analyses. Adapters were removed with Trimmomatic^46^. Cleaned reads were aligned to the human hg38 reference genome using HISAT2^47^, and read counts were generated with featureCounts^48^. Differential expression was assessed with DESeq2^49^, controlling the FDR at 0.05 with lfcThreshold = log2(1.1). We analyzed three contrasts: (1) a baseline AD signature (APP vehicle vs WT vehicle), (2) aspirin in a non-AD background (WT aspirin vs WT vehicle), and (3) aspirin in an AD background (APP aspirin vs APP vehicle).

Functional enrichment analyses were performed in R using clusterProfiler^50^ and fgsea^51^. Overrepresentation analysis (ORA) was conducted on differentially expressed genes (∣log2FC∣ ≥log2(1.1), FDR<0.05) for each contrast, with enrichment significance evaluated by hypergeometric test using all genes tested in the differential expression analysis as background. GSEA was performed on preranked gene lists for each contrast using GO, KEGG, and Reactome gene set collections, with genes ranked by Wald statistic (log2 fold change/lfcSE). For targeted neuronal analyses, human MSigDB gene sets were curated by keyword filtering of pathway names (“synapse”, “axon”, “neurotransmitter”); genes were again ranked by Wald statistic. For all enrichment analyses, multiple testing correction was performed using the Benjamini-Hochberg method, and significance was defined as FDR<0.05.

RRHO was performed using RRHO2^52^ to compare expression patterns between contrasts. Genes in each contrast were ranked by signed significance [−log_10_(*P*) × sign(log2 fold change)], and the significance of overlap between two ranked lists was assessed using hypergeometric tests across rank thresholds (step size=100). ORA was performed on discordant overlap genes (i.e., genes with significant opposite direction changes between contrasts), as described above.

## Supplementary Material

This is a list of supplementary files associated with this preprint. Click to download.

- npjSupplementaryMaterials.pdf
- SupplementaryDataFile1.xlsx

## Figures and Tables

**Table 1.** Top ten AD repurposing candidates identified in CMap queries. Drugs approved by the United States Food and Drug Administration are marked with an asterisk and their clinical indications are provided in parentheses.

GTEx brain tissues	AD-relevant GTEx tissues	All GTEx tissues	Microglia
mupirocin* (impetigo and other uncomplicated bacterial skin infections)	CAY-10618	indinavir* (human immunodeficiency virus)	anisomycin
mycophenolic-acid* (prophylaxis of organ rejection, autoimmune disease)	bufalin	isogedunin	emetine
BX-795	vidarabine* (herpes simplex keratitis)	deforolimus	homoharringtonine* (chronic myeloid leukemia)
fluticasone* (asthma, allergic rhinitis, and certain inflammatory skin conditions)	PP-30	phorbol-12-myristate-13-acetate	narciclasine
prostratin	ivermectin* (parasitic infections)	U-46619	digitoxigenin
PD-123319	rhodomyrtoxin-b	BX-795	troxipide
phenylbutyrate* (urea cycle disorders)	pentylenetetrazol	aspirin* (pain, fever, primary and secondary cardiovascular disease prevention, rheumatoid arthritis)	pyrvinium-pamoate
desoxypeganine	ingenol* (actinic keratosis)	pirinixic-acid	roscovitine
SA-63133	prostratin	ON-01910	cycloheximide
indinavir* (human immunodeficiency virus)	salubrinal	praziquantel* (parasitic infections)	isoliquiritigenin

**Table 2.** Description of matched patient cohorts used in EHR validation studies.

Clinical characteristics	VUMC (*N* = 19,413)		All of Us (*N* = 1,995)	
Aspirin	Exposed	Unexposed	Exposed	Unexposed
***N***	6,656	12,757	666	1,329
**Mean age at last follow-up (s.d.)**	77.8 (2.8)	77.9 (2.8)	77.3 (2.4)	77.3 (2.4)
Sex (%)				
Female	49.8	51.8	45.6	45.7
Male	50.2	48.2	52.4	52.5
Race (%)				
White	92.3	92.2	77.3	77.4
Black	6.8	6.8	7.1	7.3
Other	0.9	1.0	15.6	15.3
Baseline comorbidities (%)^†^				
Cardiovascular disease	25.1	21.2	28.5	28.4
Cerebrovascular disease	6.0	5.6	8.1	7.5
Rheumatoid arthritis	3.6	4.1	4.1	4.1

† Baseline comorbidities were defined as ≥1 diagnosis code recorded at or before time zero (age 65 or first encounter after 65). Code lists are provided in Supplementary Data File 1, sheets 14-16.

## Data Availability

All data are available in the main text or the supplementary materials. The AD GWAS summary statistics used in this study are publicly available at https://cncr.nl/research/summary_statistics/. The microglia eQTL summary statistics from the Microglia Genomic Atlas used in this study can be downloaded from the NIAGADS Data Sharing Service using accession number NG00105.v3. The MASHR GTEx v8 transcriptome prediction models can be downloaded from PredictDB (https://predictdb.org/categories/downloads/). Access to VUMC’s EHR database requires institutional approval and compliance with a data use agreement. Data from the *All of Us* Research Program can be accessed through the Researcher Workbench (https://workbench.researchallofus.org). The MarketScan claims data used in this study can be requested from Merative®.

## Code availability

The microglial MASHR models trained and used in this study are available on Zenodo: https://doi.org/10.5281/zenodo.18156902.
